# Optimizing the Effect of tDCS on Motor Sequence Learning in the Elderly

**DOI:** 10.3390/brainsci13010137

**Published:** 2023-01-12

**Authors:** Ensiyeh Ghasemian-Shirvan, Ruxandra Ungureanu, Lorena Melo, Kim van Dun, Min-Fang Kuo, Michael A. Nitsche, Raf L. J. Meesen

**Affiliations:** 1 Department of Psychology and Neurosciences, Leibniz Research Center for Working Environment and Human Factors, 44139 Dortmund, Germany; 2 International Graduate School of Neuroscience, Ruhr-University Bochum, 44780 Bochum, Germany; 3 Neuroplasticity and Movement Control Research Group, REVAL Rehabilitation Research Center, REVAL, Faculty of Rehabilitation Sciences, Hasselt University, 3590 Diepenbeek, Belgium; 4 Institute of Cognitive Neuroscience, Ruhr-University Bochum, 44801 Bochum, Germany; 5 University Clinic of Psychiatry and Psychotherapy and University Clinic of Child and Adolescent Psychiatry and Psychotherapy, Protestant Hospital of Bethel Foundation, University Hospital OWL, Bielefeld University, 33617 Bielefeld, Germany; 6 Movement Control and Neuroplasticity Research Group, Department of Movement Sciences, Group Biomedical Sciences, KU Leuven, 3001 Leuven, Belgium

**Keywords:** tDCS, implicit motor learning, neuroplasticity, healthy aging

## Abstract

One of the most visible effects of aging, even in healthy, normal aging, is a decline in motor performance. The range of strategies applicable to counteract this deterioration has increased. Transcranial direct current stimulation (tDCS), a non-invasive brain stimulation technique that can promote neuroplasticity, has recently gained attention. However, knowledge about optimized tDCS parameters in the elderly is limited. Therefore, in this study, we investigated the effect of different anodal tDCS intensities on motor sequence learning in the elderly. Over the course of four sessions, 25 healthy older adults (over 65 years old) completed the Serial Reaction Time Task (SRTT) while receiving 1, 2, or 3 mA of anodal or sham stimulation over the primary motor cortex (M1). Additionally, 24 h after stimulation, motor memory consolidation was assessed. The results confirmed that motor sequence learning in all tDCS conditions was maintained the following day. While increased anodal stimulation intensity over M1 showed longer lasting excitability enhancement in the elderly in a prior study, the combination of higher intensity stimulation with an implicit motor learning task showed no significant effect. Future research should focus on the reason behind this lack of effect and probe alternative stimulation protocols.

## 1. Introduction

Non-invasive brain stimulation techniques (NIBS) have been developed in the last decades as tools to monitor and modulate neuronal activity and excitability in the human brain non-invasively. Transcranial magnetic stimulation (TMS) and transcranial electrical stimulation (tES) are the main techniques in the field [1]. Transcranial magnetic stimulation applies short-lasting magnetic pulses over the head, which induce electrical fields in the brain sufficiently strong at the cortical level to induce neuronal action potentials. Also, it is suited to monitor cortical excitability if applied over regions responsive to this stimulation, such as the primary motor cortex (M1) via motor evoked potentials, or other areas via cortical evoked potentials [2]. Beyond monitoring cortical excitability, specific TMS protocols, namely repetitive TMS (rTMS), which applies trains of pulses—usually a few hundred pulses in frequencies of about 1–50 Hz—are well-suited to induce cortical excitability alterations, whose direction depend on the specific protocol and which share features of long-term potentiation (LTP) and depression (LTD) [3,4]. A specific version of TMS, paired associative stimulation, which in its classical form combines peripheral nerve electrical stimulation with cortical TMS in specific intervals, induces a kind of plasticity related to spike timing-dependent or Hebbian-like plasticity [3]. A qualitatively different stimulation protocol to modify cortical excitability is transcranial electrical stimulation (tES). This stimulation protocol induces cortical excitability alterations via subthreshold neuronal membrane polarization by a small electrical current delivered to the brain through the scalp [5,6,7]. The most relevant version of tES in connection with the present study is transcranial direct current stimulation (tDCS) [8]. tDCS modulates cortical excitability by applying a weak direct current to the brain [6] and has been extensively applied in neuroscientific and clinical research [9]. The primary mechanism of tDCS is a subthreshold modulation of the neuronal resting membrane potential towards depolarization, or hyperpolarization, through a constant weak current delivered via two or more electrodes placed on the scalp [6,9,10]. With standard stimulation protocols, anodal tDCS, which refers to a surface inward current over the target area, enhances cortical excitability. In contrast, cathodal tDCS, which refers to an outward current over the target area, reduces excitability [11,12,13]. Pharmacological studies have shown that ion channels are involved in the acute polarization effects of tDCS, which evolve immediately after the start of stimulation [11,14]. Longer lasting stimulation induces neuroplastic after-effects, which depend on the modulation of GABA and glutamatergic receptors [14,15,16]. These after-effects share features of LTP (anodal tDCS) and LTD (cathodal tDCS). Specifically, anodal tDCS reduces GABA concentration and enhances glutamatergic NMDA receptor activity, while cathodal tDCS reduces both GABA and glutamate concentrations [17,18]. For LTP to occur, glutamatergic synapses are strengthened, whereas for LTD induction, glutamatergic activity is reduced. GABA has a gating function for glutamatergic plasticity.

Neuroplasticity refers to alterations in the strength of synaptic connections as a result of environmental demands, or perturbations, such as in case of NIBS, and constitutes the foundation of cognitive processes such as learning and memory formation [19,20,21]. Numerous studies have shown that the application of NIBS improves various cognitive processes, such as learning, memory formation, and executive functions, due to synergistic–task- and stimulation-dependent changes of excitability and plasticity (Ref. review by Levasseur-Moreau, 2013 [22]). In this line, brain stimulation techniques have revealed promising results with respect to cognitive and motor rehabilitation after stroke [23,24], other neurological, and also psychiatric diseases, such as depression and schizophrenia [25,26]. Moreover, performance-reducing effects of rTMS and tDCS were also demonstrated when stimulation-induced excitability alterations antagonized respective task-dependent alterations. This was shown for reconsolidation of fear memory via low-frequency, excitability-reducing rTMS. Here, rTMS reduced memory reconsolidation. The effect was state-and timing-dependent, since it emerged only with stimulation after fear memory reactivation and within the time window relevant for reconsolidation [27]. Similarly, excitability-diminishing cathodal tDCS reduced cue-guided outcome-specific transfer in a Pavlovian to instrumental transfer task [28]. Synergistic and antagonistic stimulation in relation to task-dependent physiology has thus a specific impact on performance.

In advanced age, LTP-like neuroplasticity is reduced, which results in the deterioration of cognitive and motor abilities in the elderly [29,30,31]. Although the prevalence of physical diseases rises with age, a notable slowing of movement and reactions is also observed in otherwise healthy senior adults [32]. Older adults experience the deterioration of fine motor skills [33], a decline in reaction time task performance [34,35], and difficulties in motor coordination [36,37,38]. Moreover, a reduction in the capacity to learn new motor skills has been reported [39,40]. Such decline of motor functions and slowing of movement is caused by a multitude of age-dependent alterations, including physiological changes, structural atrophy, and neurotransmitter imbalances [41,42,43,44,45,46]. As age-related motor decline significantly impacts independent daily activities, rehabilitation techniques including non-invasive brain stimulation have been developed to modulate neuroplasticity and improve motor function in the elderly [47]. They have shown promising results with respect to cognitive and motor improvement in healthy elderly individuals and in aging-related neurological disorders [31,48,49].

Motor learning is a crucial skill in everyday life. Two main forms of motor learning can be discerned, explicit and implicit, and can be induced and modulated in experimental settings. Explicit learning refers to conscious recollection of previous experiences, while implicit learning is an unintentional, nonconscious variant of learning characterised by behavioural improvement [50]. Motor learning involves the contribution of an extended cortical-subcortical network, including the primary motor and premotor cortex, supplementary motor area, posterior parietal cortex, prefrontal areas, the striatum, and the cerebellum, with predominant contribution of specific areas, dependent on the type of task and stage of learning [50,51]. For motor sequence learning, which is the topic of this study, at the cortical level especially, M1 is relevant in the early stage of learning, while premotor and parietal areas become more important during later stages of learning, and memory consolidation [50,52,53]. In addition, at subcortical levels, the striatum and cerebellum have been proposed to be involved in motor sequence learning [50]. Motor memory acquisition requires LTP and, thus, the strengthening of learning-related synapses. Comparable to LTP induction, GABA activity decreases in the motor cortex, while glutamate is enhanced during motor learning [54,55,56]. In contrast, motor learning deteriorates when glutamatergic activity is disrupted and/or synaptic GABA activity is increased [57,58]. Thus, motor learning deficits in people of advanced age can be at least partially explained by alterations of neurotransmitters in normal aging and reduced LTP [45,59,60,61,62]. Indeed, GABA and glutamate decline and regulation impairment in the aging process have been linked to motor dysfunctions [63,64,65].

Thus, for ameliorating motor functions, and especially motor learning, induction of LTP-like plasticity could be a valuable approach. Here, tDCS might be relevant. Application of anodal tDCS over M1 has indeed been shown to enhance performance in motor sequence learning tasks [66,67,68], as well as consolidation in young adults [68,69,70,71], with effects lasting for months after intervention in some studies [72,73]. However, some heterogeneity of results was also noted [52,74,75], which might be at least partially caused by intervention protocol differences. One of these is the timing-dependency of tDCS effects on motor learning. It was thus shown that the intervention should be conducted during the actual motor learning process to be effective, in order to make use of synergistic stimulation- and learning-dependent plasticity induction [58].

Likewise, tDCS studies to improve motor learning in the elderly revealed partially heterogeneous results. In some studies, tDCS in combination with sequence motor learning enhanced learning and improved consolidation in the elderly [76,77,78,79], as well as in patients with motor deficits and impaired movement abilities [80,81,82,83,84]. However, other studies show no enhancement of motor learning [47] or motor improvement by tDCS after stroke [83,85]. One reason for heterogeneous results beyond methodological causes, including the use of different intervention protocols, outcome measures, and target groups in respective studies, might be inter-individually heterogeneous brain structures and connectivity, as well as neuromodulator and neurotransmitter alterations in advanced age. Loss of grey matter volume with aging [86] causes a reduction of cognitive and motor performance due to the disintegration of cerebral networks [86]. These structural alterations also result in larger variability of the distribution of stimulation-induced electric fields [87], which might lead to heterogeneous tDCS effects. Studies in elderly populations have moreover revealed that induction of LTP-like plasticity by tDCS declines with advancing age, potentially requiring a higher intensity or longer duration of stimulation [88,89,90,91,92]. Thus, it might be speculated that enhancing the tDCS dosage strengthens the impact of this intervention on motor learning in the elderly. However, few studies have aimed to develop optimally suited tDCS protocols to prevent motor function deterioration in the elderly population.

Prior studies have shown that enhanced anodal tDCS intensity and duration (i.e., 3 mA-20 min, 3 mA-30 min) can induce larger and longer lasting LTP-like plasticity in the elderly [93]. Therefore, in this study, we systematically titrated the intensity of anodal tDCS over M1 during a motor sequence learning task in elderly participants, aiming for an optimized stimulation protocol for functional improvement. In a double-blind, sham-controlled, cross-over design, we applied three stimulation intensities (1, 2, and 3 mA) in combination with an implicit motor sequence learning task. We hypothesized that higher intensities of tDCS will result in greater improvement of motor learning. Establishing an effective stimulation protocol would potentially allow for motor function improvements in the healthy elderly population or patients with motor skill disorders.

## 2. Materials and Methods

### 2.1. Participants

A Power analysis (G*Power 3.1.9.4, Franz Faul, Universität Kiel, Germany), for estimating the required sample size, conducted based on a medium critical effect size (0.25) and critical alpha and beta-errors of 0.05, resulted in a sample size of 23 participants using an ANOVA as the primary statistical test. In order to account for dropouts, we added 2 more participants. Therefore, twenty-five healthy, right-handed, non-smoking elderly volunteers above 65 years (14 females; 11 males; mean age 72.13, SD ± 5.91) were recruited. The experiment was conducted by the same experimenter in Dortmund (Germany) and later in Hasselt (Belgium) using the identical experimental protocol. A physician examined all volunteers for exclusion criteria for non-invasive electrical brain stimulation [94,95]. Handedness was examined with the Edinburgh Handedness Inventory [96], and cognitive state was evaluated with the Montreal Cognitive Assessment test (MoCA) [97]. Volunteers participating in the study signed an informed consent form and received financial compensation. This study was approved by the ethics committee of Leibniz Research Centre for Working Environment and Human Factors (IfADo) and by the Committee for Medical Ethics of Hasselt University and is in accordance with the Declaration of Helsinki [98].

### 2.2. Transcranial Direct Current Stimulation (tDCS)

Prior to stimulation, a topical anaesthetic cream (EMLA^®^, 2.5% lidocaine, 2.5% prilocaine (Dortmund, Aspen Pharma trading Limited, Dublin, Irland) or lidocaine HCL 2.5% buffered cetomacrogol cream (Hasselt, Aa.Pharma Oud-Turnhout, Hasselt, Belgium) was applied over the stimulation areas to ensure sufficient blinding of the participants [99]. In randomized order, all participants received 3 sessions of anodal stimulation at 1, 2, or 3 mA intensity, and one sham stimulation session. tDCS was delivered by a battery-powered constant current stimulator (neuroCare, Ilmenau, Germany) through a pair of carbonated rubber electrodes embedded in saline-soaked sponges (35 cm^2^). The anode was fixed over M1 (C3, according to the international 10–20 EEG system), and the cathode was placed contralaterally over the right supraorbital region. The duration of stimulation was adjusted to the duration of motor task performance and controlled via an external trigger (flip-flop mode) with a 30 s ramp-up at the start of the stimulation, and a 30 s ramp-down at the end of the stimulation. For sham stimulation, 1 mA was applied for 30 s (ramp-up and down duration was the same as for other stimulation protocols), followed by 0 mA until task completion. The interval between sessions was at least one week to avoid carry-over effects of the stimulation.

### 2.3. Serial Reaction Time Task (SRTT) as a Measure of Implicit Motor Sequence Learning

A custom-made response box with four response keys anatomically aligned to the right hand was used to record performance. The four fingers involved in performance were the index finger for the first button, the middle finger for the second button, the ring finger for the third button, and the little finger for the fourth button. Four horizontal lines representing the keys were presented on the computer screen. Participants were instructed to press the corresponding key with the correct finger as fast and accurately as possible when a stimulus (white dot) appeared above the corresponding line. The next trial appeared 500 ms following a button press, independent of a correct or incorrect response.

The test was performed on two consecutive days. On the first day, the task was accompanied by tDCS (main-SRTT [M-SRTT]), consisting of 8 blocks, each with 120 trials. Blocks 1 and 6 were random blocks, in which the trials were presented in a pseudo-random order. Blocks 2–5 and 7–8 displayed a 12-trial sequence of stimuli repeated 10 times in each block, which prompted implicit learning [100]. Blocks were separated by self-paced breaks and participants were not informed about the sequences. On the second day, a short form of the SRTT was performed (recall-SRTT [R-SRTT]), consisting of three blocks: one random block followed by two sequence blocks. In order to control for use-dependent sequence learning over sessions, four different sequences were designed for the main SRTT. Thus, participants performed a different sequence in each session. The sequence at the recall day was the same as the main SRTT session performed on the day before. Following the last session, participants were asked if they noticed any repeating/pattern of the stimuli.

### 2.4. Experimental Procedure

**Figure 1:**
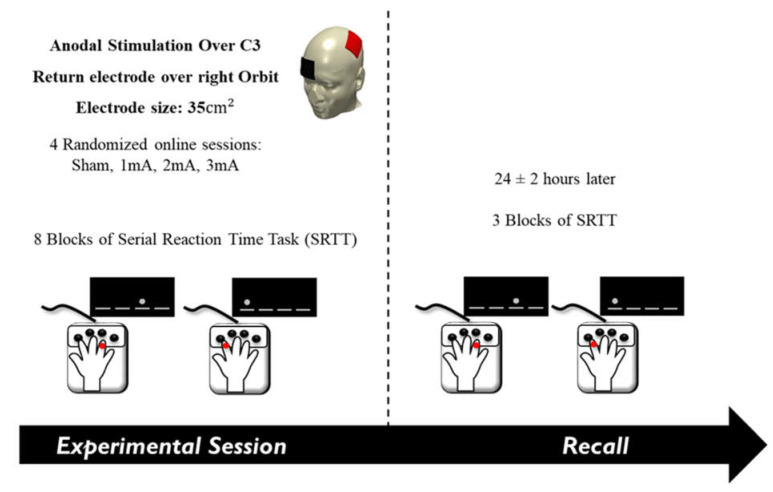
Course of the experiment. The anode electrode was placed over the left primary motor cortex (C3) and the cathode over the right supraorbital area. In a random order, sham, 1, 2, or 3 mA of tDCS was delivered in separate sessions while participants performed the serial reaction time task. Participants performed the recall session the following day.

The study was performed in a cross-over, double-blind, randomized design. Both participants and experimenter were blinded with respect to the tDCS condition. Every session started with the identification of the C3 position (according to the 10–20 International EEG system). The anaesthetic cream was then applied to the scalp where the tDCS electrodes would be placed. tDCS and M-SRTT started 20 min later, allowing the anaesthetic effect of the cream to set in. Participants were seated in front of a computer screen at eye level, with the table and chair adjusted to each individual. The performing hand of each participant was covered by a box designed specifically for the experiment to avoid any distractions (e.g., by visual checking the button to be pressed). Before starting the main part of the experiment, participants were asked to perform a 20-trial random block of SRTT to familiarize themselves with the task and to determine the best position for their hands. The tDCS and M-SRTT were synchronized so that the first trial appeared after the 30 s ramp-up of tDCS. The stimulation stopped after the last trial, which prompted tDCS to ramp-down. Next, the electrodes were removed, and participants were asked to fill in a questionnaire that evaluated blinding and side effects of tDCS during and after stimulation [101,102]. The questionnaire contained (1) participants’ guess of the applied tDCS intensity (0, 1, 2, and 3 mA) and (2) rating scales for the presence and perception of visual phenomena, itching, tingling, burning, and pain during stimulation, and (3) rating scales for the presence and amount of skin redness, headache, fatigue, concentration difficulties, nervousness, and sleep problems within 24 h following stimulation. The third question was asked the following day. The side effects were rated on a numerical scale from zero (‘none’) to five (‘extremely strong’). The recall session was performed 24 ± 2 h after the main session (Figure 1., Course of the experiment).

### 2.5. Data Analysis and Statistics

The individual mean reaction time (RT) and standard deviation (SD) of correct responses was calculated for each block of the experimental condition and for each participant, separately. RTs above or below 3 standard deviations (SD) were discarded in each block. Mean RTs were normalised to block 1 for each participant, in each stimulation condition, separately.

#### 2.5.1. Effect of tDCS on M-SRTT

To rule out the possibility of baseline RT differences between the tDCS conditions, the respective absolute RTs of block 1 were compared through a one-way ANOVA. Separate repeated measures ANOVAs were conducted for reaction time and accuracy, with the independent variables ’Condition’ (4 levels) and ‘Block’ (8 levels) and dependent variables, including absolute and normalized RTs, number of errors, and variability (SDs of absolute RTs).

#### 2.5.2. Effect of tDCS on R-SRTT

To explore the effect of tDCS on recall SRTT, a repeated measures ANOVA was calculated with the independent variables ‘Condition’ (4 levels) and ‘Block’ (3 levels) and dependent variables, including both absolute and normalized RTs, number of errors, and variability of the recall session.

#### 2.5.3. Assessment of tDCS Side Effects and Blinding

A Chi-square test was conducted to identify whether participants correctly guessed tDCS intensities. The presence of side effects during and after tDCS was analysed separately using a repeated measures ANOVA with ‘Condition’ (4 levels) as the within-subject factor and rating scores (0–5) as the dependent variable.

For the ANOVAs, Mauchly’s test of sphericity was conducted, and the Greenhouse–Geisser correction was applied when necessary. The critical significance level was set at *p* ≤ 0.05. In the case of significant ANOVA results, exploratory post-hoc Fisher’s Least Significant Difference (LSD) tests were conducted. Statistical analyses were performed with SPSS (IBM Corp. Version 27.0, Armonk, New York, USA).

## 3. Results

All participants tolerated the stimulation well. One participant was excluded from all data analyses due to poor performance in one session with an error rate of 50%.

The results of the MoCA test were in the normal range for all participants (mean ± SD: 26.21 ± 2.37). The duration of tDCS was adjusted to the length of the task, which was on average 21.79 ± 1.17 min (mean ± SD). There was no significant difference of stimulation duration between the conditions (F_(2.279, 52.409)_ = 0.498, *p* = 0.635, $\eta_{p}^{2}=0.021$). The average of discarded trials for the M-SRTT was 5.33% ± 3.81 (mean ± SD) and, for the R-SRTT, it was 6.78% ± 11.19. Regarding sequence awareness, only 3 of the 24 participants reported that they did not notice a sequence.

### 3.1. Effect of tDCS on M-SRTT

**Figure 2:**
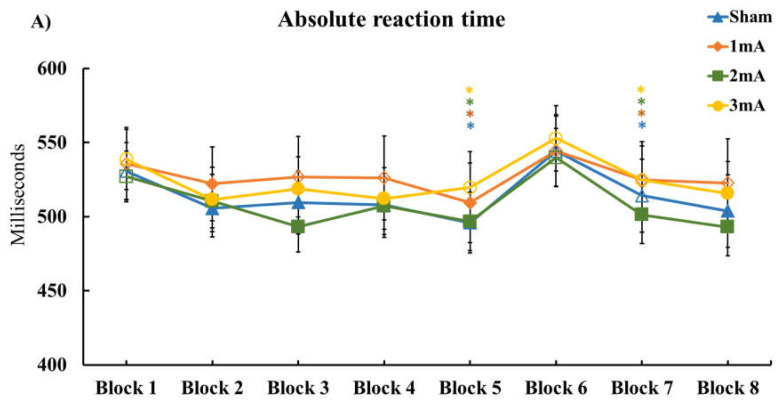

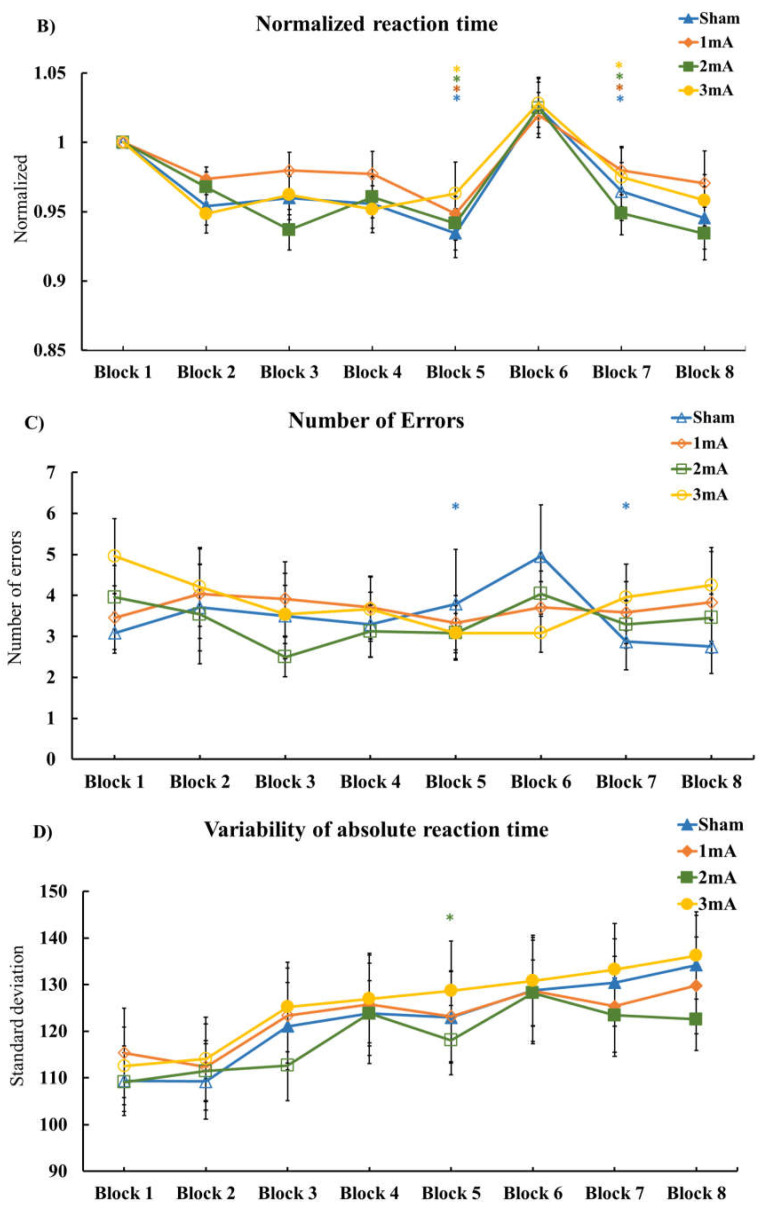
Results of the M-SRTT. The graphs show results for (**A**) absolute RT, (**B**) normalized RT, (**C**) number of errors, and (**D**) variability of absolute RT across different tDCS protocols. Filled symbols indicate a significant difference compared to block 1 in each condition. Asterisks (⁎) refer to significant reaction time differences between block 5–6 (sequence-random block) or block 6–7 (random-sequence block). Error bars represent standard error of means.

The one-way repeated measures ANOVA results of M-SRTT comparing absolute RTs of block 1 for each tDCS condition showed no significant difference (F_(3, 69)_ = 0.300, *p* = 0.825, $\eta_{p}^{2}=0.013$). The results of the repeated measures ANOVA for absolute RT values revealed a significant main effect of ‘Block’ (F_(2.521, 57.977)_ = 13.486, *p* < 0.001, $\eta_{p}^{2}=0.370$) but not ‘Condition’ effect (F_(3, 69)_ = 0.827, *p* = 0.484, $\eta_{p}^{2}=0.035$), nor a significant ‘Condition’ × ‘Block’ interaction (F_(8.790, 202.169)_ = 1.290, *p* = 0.245, $\eta_{p}^{2}=0.053$) (Table 1 (A) Effect of tDCS on M-SRTT; Figure 2A Results of the M-SRTT).

Similarly, the respective ANOVA for normalized RTs showed a significant main effect of ‘Block’ (F_(2.502, 57.539)_ = 14.996, *p* < 0.001, $\eta_{p}^{2}=0.395$) but not ‘Condition’ (F_(3, 69)_ = 0.572, *p* = 0.635, $\eta_{p}^{2}=0.024$), nor a ‘Condition’ × ‘Block’ interaction (F_(8.982, 206.595)_ = 1.192, *p* = 0.302, $\eta_{p}^{2}=0.049$) (Table 1 (B) Effect of tDCS on M-SRTT) (Figure 2B Results of the M-SRTT).

Furthermore, the ANOVA results for ‘Errors’ showed no significant effects of the main factors ‘Block’ (F_(2.639, 60.695)_ = 0.637, *p* = 0.575, $\eta_{p}^{2}=0.027$) and ‘Condition’ (F_(1.785, 41.044)_ = 0.408, *p* = 0.645, $\eta_{p}^{2}=0.017$), nor the ‘Condition’ × ‘Block’ interaction (F_(3.763, 60.695)_ = 1.286, *p* = 0.282, $\eta_{p}^{2}=0.053$) (Table 1 (C) Effect of tDCS on M-SRTT) (Figure 2C Results of the M-SRTT).

The ANOVA results of M-SRTT ‘Variability’ also showed a significant main effect of ‘Block’ (F_(2.742, 63.069)_ = 11.787, *p* < 0.001, $\eta_{p}^{2}=0.339$) but not ‘Condition’ (F_(3, 69)_ = 0.538, *p* = 0.658, $\eta_{p}^{2}=0.023$), nor the ‘Condition’ × ‘Block’ interaction (F_(9.399, 216.171)_ = 0.743, *p* = 0.788, $\eta_{p}^{2}=0.031$) (Table 1 (D) Effect of tDCS on M-SRTT) (Figure 2D Results of the M-SRTT).

Exploratory post-hoc reaction time tests in the 1 mA stimulation condition showed no significant differences between sequence blocks relative to block 1 (all post-hoc test results, including degrees of freedom, t-values, *p*-values, and effect sizes, are available in the Supplemental Material, Tables S1–S12), except for blocks 2 and 5. For 2 mA, all sequence blocks differed significantly from block 1. In addition, except for blocks 5 and 7, all sequenced blocks in the 3 mA tDCS condition differed significantly from block 1. Also, with the exception of block 7, all sequence blocks in the sham condition were significantly different from block 1. Furthermore, exploratory post-hoc LSD showed a significant difference between block 6 (random trials) and blocks 5 and 7 (sequence trials), demonstrating that motor sequence learning took place in all tDCS conditions.

Moreover, exploratory post-hoc LSD for error counts showed a significant decrement of errors in the 3 mA tDCS condition for blocks 3 and 5 compared with block 1. There was also a significant increment in the number of errors in block 6 in comparison to blocks 5 and 7 in the sham condition.

Regarding variability of absolute reaction time, the exploratory post-hoc test revealed a significant increase of variability of blocks 4 and 6–8, as compared with block 1, for the 1 and 2 mA conditions. Moreover, except for block 2, variability of all blocks in the 3 mA and sham conditions were significantly increased when compared to block 1.

### 3.2. Effects of tDCS on R-SRTT

**Figure 3:**
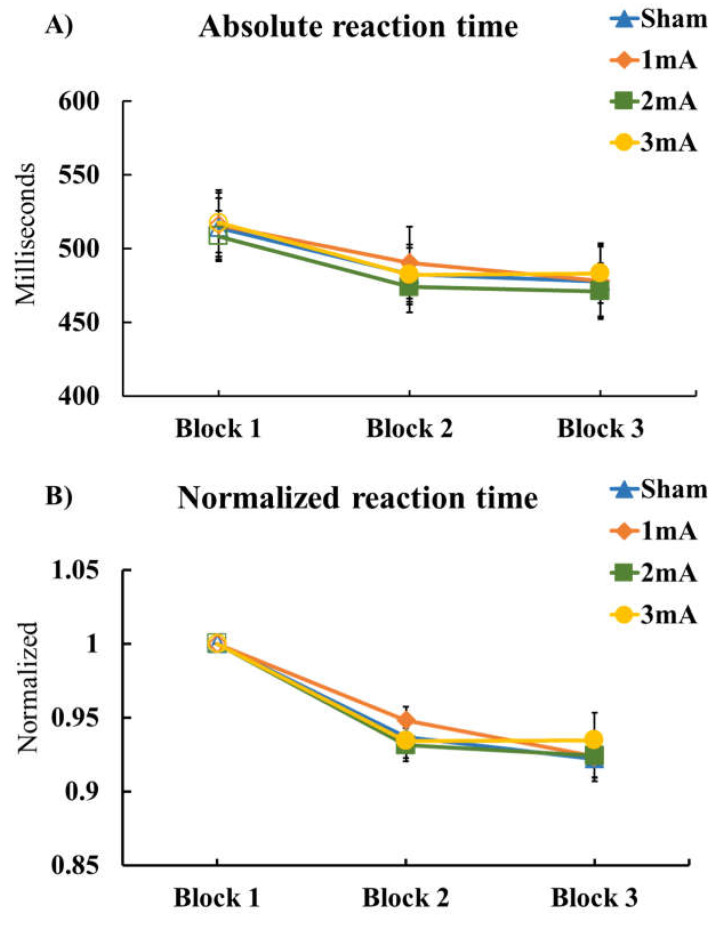

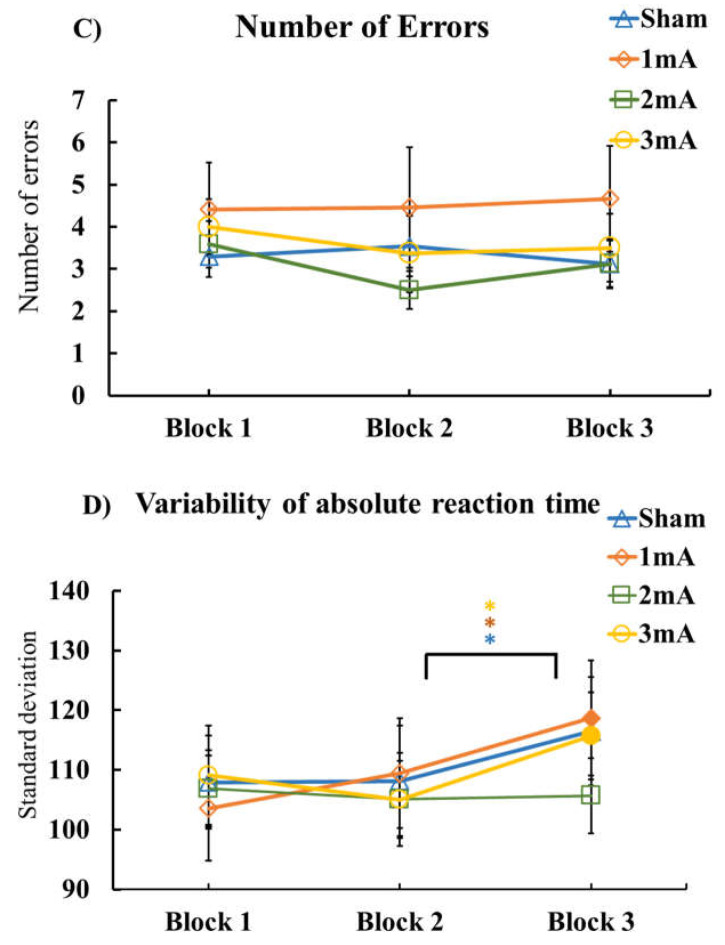
Results of R-SRTT. The graphs show (**A**) absolute reaction time, (**B**) normalized reaction time, (**C**) number of errors, and (**D**) variability of absolute reaction time of R-SRTT performance 24 h after each tDCS protocol. Filled symbols indicate a significant difference in each block compared to block 1. Asterisks (⁎) refer to significant differences of block 2 to block 3 (both sequence blocks). Error bars represent standard error of means.

The ANOVA conducted on R-SRTT comparing absolute RTs of block 1 between tDCS conditions showed no significant difference (F_(3, 69)_ = 0.276, *p* = 0.843, $\eta_{p}^{2}=0.012$). The respective repeated measures ANOVA of absolute RTs revealed a significant main effect of ‘Block’ (F_(1.554, 35.751)_ = 42.761, *p* < 0.001, $\eta_{p}^{2}=0.650$) but not ‘Condition’ (F_(3, 69)_ = 0.351, *p* = 0.789, $\eta_{p}^{2}=0.015$) nor the ‘Condition’ × ‘Block’ interaction (F_(3.773, 86.780)_ = 0.517, *p* = 0.713, $\eta_{p}^{2}=0.022$) (Table 2 (A) Effect of tDCS on R-SRTT; Figure 3A Results of R-SRTT).

In addition, the ANOVA conducted for normalized RTs showed a significant main effect of ‘Block’ (F_(1.397, 32.128)_ = 41.488, *p* < 0.001, $\eta_{p}^{2}=0.643$) but not ‘Condition’ (F_(3, 69)_ = 0.139, *p* = 0.936, $\eta_{p}^{2}=0.006$), nor the ‘Condition’ × ‘Block’ interaction (F_(3.634, 83.587)_ = 0.508, *p* = 0.712, $\eta_{p}^{2}=0.022$) (Table 2 (B) Effect of tDCS on R-SRTT; Figure 3B Results of R-SRTT).

The ANOVA results for ‘Errors’ showed no significant main effects of ‘Block’ (F_(2, 46)_ = 0.666, *p* = 0.517, $\eta_{p}^{2}=0.028$) or ‘Condition’ (F_(1.779, 40.912)_ = 1.118, *p* = 0.331, $\eta_{p}^{2}=0.046$), nor the ‘Condition’ × ‘Block’ interaction (F_(3.584, 82.437)_ = 0.598, *p* = 0.647, $\eta_{p}^{2}=0.025$) (Table 2 (C) Effect of tDCS on R-SRTT; Figure 3C Results of R-SRTT).

For R-SRTT variability of absolute reaction time, the ANOVA showed a significant main effect of ‘Block’ (F_(1.565, 36.001)_ = 6.024, *p* < 0.009, $\eta_{p}^{2}=0.208$) but not ‘Condition’ (F_(3, 69)_ = 0.472, *p* = 0.703, $\eta_{p}^{2}=0.020$). There was a significant effect of the ‘Condition’ × ‘Block’ interaction (F_(6, 138)_ = 2.643, *p* = 0.019, $\eta_{p}^{2}=0.103$) (Table 2 (D) Effect of tDCS on R-SRTT; Figure 3D Results of R-SRTT).

Additionally, exploratory post-hoc LSD for RTs of the R-SRTT showed a significant decrement of RTs between block 1 (random trials) and block 2 and 3 (sequence trials) in all conditions.

**Figure 4:**
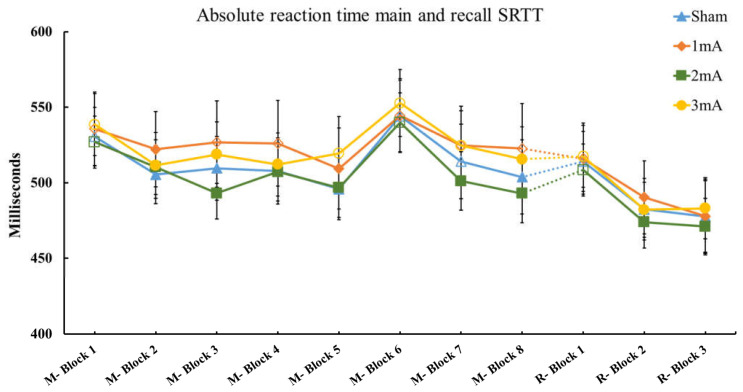
Absolute reaction time M-SRTT and R-SRTT. The graph displays the absolute reaction time of main and recall SRTT in all conditions.

Furthermore, the post-hoc LSD showed a significant enhancement of variability between block 1 and block 3 in all conditions, except the 2 mA stimulation. Moreover, RT variability in the 1 mA, 3 mA, and sham conditions were significantly higher in block 3, as compared with block 2. Figure 4 presents the results of both absolute M-SRTT and R-SRTT for all conditions.

### 3.3. Assessment of tDCS Side Effects and Blinding Efficacy

The data points of the guessed versus actually received stimulation intensities are presented in Table 3 (Data points of guessed versus received tDCS intensity). The Chi-square test revealed no significant heterogeneity (χ^2^ Value = 3.042, F (9), *p* = 0.963), meaning blinding was successful.

Participants’ ratings for the presence and intensity of side effects during and within 24 h after stimulation are listed in Table 4 (Side effect ratings). The ANOVAs showed no significant difference in the side effect ratings during or 24 h following stimulation between conditions (Table 5. Side effect ratings of the participants, ANOVA results).

## 4. Discussion

All participants tolerated the intervention well, and blinding was successful.

As the results of the M-SRTT demonstrate, reaction time in sequence blocks decreased over time in comparison to random blocks in all stimulation conditions. Thus, motor learning took place; however, it did not significantly differ between intervention conditions. Only under 2 mA anodal stimulation, reaction time was significantly reduced in all sequence blocks compared to the random block 1, which might be interpreted as a trend for more stable motor learning in this condition. Furthermore, reaction times in block 5 and 7 (sequence) in relation to block 6 (random) were significantly reduced in all conditions, confirming motor sequence learning effects during all interventions. Also, for the sequence learning-specific analysis, the tDCS effects of different intensities did not differ. Regarding the number of errors and reaction time variability, the stimulation conditions also did not differ in the M-SRTT.

Likewise, the R-SRTT revealed a significant reduction of reaction time in the sequence blocks but no differences between stimulation conditions. This suggests that memory consolidation also took place in all stimulation conditions. With respect to errors, no difference was observed between conditions, but RT variability showed a significant increase when comparing block 3 (sequence) to block 2 (sequence) and block 3 (sequence) to block 1 (random) under sham, 1 mA, and 3 mA conditions. Hereby, the higher variability of block 3 might be caused by the fact that, for a well-learned sequence, the RT difference between well-performed and not well performed trials is expected to be larger than in a less well-learned sequence and within a random stimulus block where sequence memory does not interfere.

These results differ from most previous studies, which have shown that implicit motor sequence learning is enhanced by anodal tDCS applied to M1 [103]. Improvement of online reaction time [66,67,68,72,104,105], consolidation and retention [68,69,70,71], and reduction of the number of errors [71] has been documented in young adults. Nevertheless, some studies combining anodal tDCS over M1 with the serial reaction time task in young volunteers have shown contrasting results. In an offline tDCS study, no significant improvement of learning was observed after anodal stimulation with 1 mA for 10 min [74], suggesting that synchronous stimulation and task performance might be relevant for the effects. Effects of tDCS on learning-related plasticity should be stronger and more specific to synchronous learning and stimulation when task-related neurons are activated by the learning process and stimulation. Moreover, in another study investigating different intensities of stimulation on explicit motor sequence learning task performance, no difference was observed for either 1 mA or 1.5 mA stimulation compared to sham in the main experimental session, but, compared to sham stimulation, a significant improvement in the 1.5 mA condition in the retention session was revealed [70]. This delayed effect might be because tDCS-induced LTP-like plasticity should strengthen task-relevant neuronal connections for prolonged periods of time. These effects might be uncovered with a delay when only learning-related plasticity already starts to vanish [58]. These intervention protocol-dependent missing effects do not apply to the present study’s results, because tDCS was applied during learning, and we re-tested SRTT performance in a consolidation session.

Studies investigating motor sequence learning tasks with concurrent tDCS in elderly groups have also shown heterogeneous results. Reaction time and error decrements in motor sequence learning tasks under tDCS in the elderly have been reported [76,78,79]. Some of these studies have even investigated the effects of multi-session anodal tDCS in the elderly, which led to extended improved motor performance over several months after intervention [65]. Others, though, have reported no effect of tDCS on motor sequence learning [47,106], which might be partially due to methodological specifics, as outlined above, but these do not fully explain the missing effects in some studies, including the present one.

Results regarding consolidation in the present study revealed that implicit motor sequence learning was retained after 24 h regardless of the stimulation protocol. In contrast, other studies showed that motor memory consolidation was enhanced by tDCS in young [69,72] and elderly adults [79]. However, it should be emphasized that the aforementioned studies did not use the same methodology (e.g., stimulation duration, intensity, and timing-dependency of tDCS on motor learning) as the present study, and these effects on consolidation emerged along with improved learning under tDCS, which was not the case in the present study. This might explain the missing effects of tDCS on consolidation in the present study.

While methodological disparities might partially explain the heterogeneous effects between studies, these do not deliver a complete explanation. Therefore, we propose that age-dependent alterations of brain functions contribute and explain the missing effects of tDCS in the present study. Differences in LTP-like plasticity induction between age groups have been previously reported, which showed reduced LTP-like plasticity induction by anodal tDCS in the elderly [88,90]; however, intensified protocols of 3 mA-20 min and 3 mA-30 min of anodal tDCS, as applied in the present study, resulted in enhanced and longer-lasting LTP-like plasticity in this age group [93]. This led to the expectation that the combination of a motor sequence learning task with intensified stimulation would result in improved effects.

It might be the case that age-dependent alterations at the microscale level contributed to the missing effect of tDCS on SRTT performance in the present study. Imaging studies show that the reduction of GABA concentration in M1 plays an important role during the acquisition of a sequence of movements [58,107,108]. Moreover, decreased GABA and enhanced glutamate concentration under anodal tDCS have been shown to be necessary for LTP-like plasticity induction, that, as previously explained, shares a mechanism of action with motor learning [18,72,105,109,110]. The likely foundation of motor learning, including the acquisition and retention of skills, is use-dependent LTP-like plasticity, which is regulated by NMDA receptor activity and GABA [111]. In this connection, it is worth mentioning that dysregulation of neurotransmitters and neuromodulators in advanced age has been documented in animal and human studies. This includes a significant reduction of GABA concentration in M1 in older adults compared to those of younger age [112]. Likewise, glutamate concentration in the brain declines with age [62,113,114,115]. These GABA and glutamate alterations may potentially limit motor performance improvements by tDCS [116,117,118]. A fine-tuned excitation/inhibition (E/I) balance is required to induce the plasticity of task-relevant neurons. Reduced GABA in older age will compromise respective inhibition, which will be further be enhanced by tDCS [16], and thus, likely reduce de-activation of task-irrelevant neurons. Since the tDCS effects are furthermore not restricted to task-relevant neurons, this will likely result in a noisy LTP, which might not be efficient for improved learning. In accordance, an MRS motor sequence learning study in the elderly showed no reduction of GABA levels in the sensorimotor cortex during motor training, which, in connection to the general GABA reduction in the elderly, hints to a bottom effect [106]. It could be thus speculated that relative glutamatergic over-activation plays a role in these missing effects; therefore, applying cathodal tDCS to reduce this might cause enhanced performance in the elderly population. Such an effect has been shown to aid performance improvement in a ’noisy’ learning task in young adults; however, too high diffuse LTP, as induced by anodal tDCS, was detrimental [119]. This explanation is speculative at present and has to be substantiated in future studies.

Age-dependent alterations of brain structure and function at the macroscale level might have also contributed to the missing effects of anodal tDCS in the present study. As mentioned earlier, structural decline of the central nervous system in advanced age, such as grey and white matter atrophy, might reduce motor and cognitive performance [106,120,121] and also tDCS effects. The latter might be due to an enhanced stimulation electrode-to-brain distance [122]. With respect to functional connectivity, older adults exhibit weaker functional connectivity between brain regions within the core functional network, which compromises this network’s efficacy and performance. Since tDCS induces network effects [123], this might also reduce the impact of tDCS on motor learning. However, the elderly show a more distributed connectivity with other cerebral areas [124]. This was explained by compensatory activity, meaning that this additional activity is required to keep performance at a similar level despite reduced functionality of the core network [125]. It might thus be speculated that the primary motor cortex is already at its maximum level of functionality for the learning process without additional interventions; thus, its efficacy cannot be enhanced further by direct stimulation. Therefore, it might be speculated that compensatory areas are more promising intervention targets for performance improvement [124]. Nevertheless, this needs to be substantiated in future studies.

Taken together, enhanced stimulation intensity over the motor cortex did not lead to a relevant motor learning improvement in elderly participants in the present study. Thus, the application of tDCS for performance improvement in the elderly might be more challenging than in young adults. This might be caused by an aging-dependent LTP-like plasticity decline due to various alterations of the aging brain at micro- and macroscale levels, including neurotransmitter decline, brain atrophy, and altered network connectivity. Therefore, adapted stimulation protocols, including re-establishing compromised cortical inhibition and stimulation of different network components—such as those activated only in higher age to compensate for reduced functionality of the motor core network have to be developed and should be probed in future studies.

### Limitations and Future Directions

There are several limitations that warrant further discussion. While session order was randomized in this crossover study and had no significant effect on the outcome, a counter-balanced design would have improved the validity of the results further by ruling out order effects more definitely. With consideration of the excluded participant, an additional sample of 27 participants would have had to be recruited for a balanced order. Given this present limited timeline and the pandemic, this was not feasible. A potential ceiling effect might have also affected the reduction in reaction time as participants performed multiple sessions of the task.

Finally, some studies have reported that stimulating the cerebellum induces better motor memory acquisition [71,126]. Exploring the effect of tDCS on other brain regions or combining it with imaging techniques in the elderly group could thus improve our mechanistic knowledge and might lead to advanced protocols.

## 5. Conclusions

This study explored the titration of anodal tDCS intensities (1, 2, and 3 mA) over M1 to optimize implicit motor learning performance in older adults. Our results show that implicit motor learning, as well as consolidation, occurred regardless of stimulation conditions. Exploration of the reasons for this null effect and respective adaptation of intervention protocols should be the subject of future studies. Identification of the optimal protocol for improving motor learning in the healthy elderly might improve performance in older, healthy adults and also enhance the efficacy of tDCS in neurorehabilitation patients. It is important to state that a one-to-one transferability of these effects to other cortical areas and patient populations should not be presumed, due to the state-dependency of tES effects, anatomical differences, and differences of neuromodulator activities and cortical excitability between healthy humans and respective patients.

## Figures and Tables

**Table 1:** Effect of tDCS on M-SRTT. Results of the repeated measures ANOVAs for the M-SRTT: (A) absolute RTs, (B) normalized RTs, (C) number of errors, and (D) variability of absolute RTs are shown. Asterisks indicate significant results (*p* < 0.05). d.f. = degrees of freedom, $\eta_{p}^{2}$ = partial eta squared.

	Factor	d.f., Error	F Value	$\eta_{p}^{2}$	*P* Value
(A) Absolute RT	Condition	3, 69	0.827	0.035	0.484
	Block	2.521, 57.977	13.486	0.370	<0.001 *
	Condition × Block	8.790, 202.169	1.290	0.053	0.245
(B) Normalized RT	Condition	3, 69	0.572	0.024	0.635
	Block	2.502, 57.539	14.996	0.395	<0.001 *
	Condition × Block	8.982, 206.595	1.192	0.049	0.302
(C) Errors	Condition	1.785, 41.044	0.408	0.017	0.645
	Block	2.639, 60.695	0.637	0.027	0.575
	Condition × Block	3.763, 86.548	1.286	0.053	0.282
(D) Variability	Condition	3, 69	0.538	0.023	0.658
	Block	2.742, 63.069	11.787	0.339	<0.001 *
	Condition × Block	9.399, 216.171	0.743	0.031	0.788

**Table 2:** Effect of tDCS on R-SRTT. Results of the mixed-model ANOVAs conducted for R-SRTT: (A) absolute RTs, (B) normalized RTs, (C) number of errors, and (D) variability of absolute RTs. Asterisks indicate significant results (*p* < 0.05). d.f. = degrees of freedom, $\eta_{p}^{2}$ = partial eta squared.

	Factor	d.f., Error	F Value	$\eta_{p}^{2}$	*P* Value
(A) Absolute RT	Condition	3, 69	0.351	0.015	0.789
	Block	1.554, 35.751	42.761	0.650	<0.001 *
	Condition × Block	3.773, 86.780	0.517	0.022	0.713
(B) Normalized RT	Condition	3, 69	0.139	0.006	0.936
	Block	1.397, 32.128	41.488	0.643	<0.001 *
	Condition × Block	3.634, 83.587	0.508	0.022	0.712
(C) Errors	Condition	1.779, 40.912	1.118	0.046	0.331
	Block	2, 46	0.666	0.028	0.517
	Condition × Block	3.584, 82.437	0.598	0.025	0.647
(D) Variability	Condition	3, 69	0.472	0.020	0.703
	Block	1.565, 36.001	6.024	0.208	0.009 *
	Condition × Block	6, 138	2.643	0.103	0.019*

**Table 3:** Data points of guessed versus received tDCS intensity. After each session, participants filled in a questionnaire asking them to guess the intensity of applied tDCS (0, 1, 2, 3 mA). The table contrasts actually applied (rows) with perceived stimulation intensities (columns).

Data Points		Guessed Stimulation Intensity				Total Data Points
		Sham	1 mA	2 mA	3 mA	
Real tDCS Intensity	Sham	14	6	1	4	25
	1 mA	16	4	3	2	25
	2 mA	13	6	2	4	25
	3 mA	13	6	3	3	25

**Table 4:** Side effect ratings included visual phenomena, itching, tingling, and pain during stimulation, and skin redness, headache, fatigue, difficulty in concentration, nervousness, and sleep problems within 24 h after stimulation. The presence and intensity of side effects were rated on a numerical scale from zero (feeling nothing) to five (extremely strong feeling). Data are presented as mean ± SD.

Side Effects	Sham	1 mA	2 mA	3 mA
Visual Phenomena	0.08 ± 0.40	0.04 ± 0.20	0.37 ± 1.27	0.12 ± 0.33
Itching	0.00 ± 0.00	0.00 ± 0.00	0.12 ± 0.44	0.25 ± 0.67
Tingling	0.04 ± 0.20	0.04 ± 0.20	0.04 ± 0.20	0.16 ± 0.48
Burning	0.04 ± 0.20	0.12 ± 0.61	0.20 ± 0.65	0.20 ± 0.65
Pain	0.00 ± 0.00	0.00 ± 0.00	0.00 ± 0.00	0.12 ± 0.44
Redness	0.00 ± 0.00	0.16 ± 0.56	0.08 ± 0.40	0.08 ± 0.40
Headache	0.00 ± 0.00	0.12 ± 0.61	0.08 ± 0.40	0.04 ± 0.20
Fatigue	0.20 ± 0.50	0.12 ± 0.44	0.16 ± 0.48	0.08 ± 0.28
Concentration	0.08 ± 0.28	0.08 ± 0.28	0.20 ± 0.41	0.16 ± 0.38
Nervousness	0.00 ± 0.00	0.00 ± 0.00	0.00 ± 0.00	0.04 ± 0.20
Sleep Problems	0.00 ± 0.00	0.04 ± 0.20	0.08 ± 0.40	0.04 ± 0.20

**Table 5:** Side effect ratings of the participants, ANOVA results. The results showed no significant effect for any of the side effects during or within 24 h following stimulation.

	Side Effects	d.f., Error	F Value	$\eta_{p}^{2}$	*P* Value
During Stimulation	Visual phenomena	1.216, 27.967	1.174	0.049	0.300
	Itching	1.692, 38.922	2.213	0.088	0.094
	Tingling	1.000, 23.000	1.865	0.075	0.185
	Burning	1.690, 38.866	1.105	0.046	0.333
	Pain	1.000, 23.000	1.865	0.075	0.185
After Stimulation	Redness	1.428, 32.840	0.793	0.033	0.422
	Headache	1.000, 23.000	1.000	0.042	0.328
	Fatigue	2.284, 52.542	1.264	0.052	0.294
	Concentration	3, 69	1.084	0.045	0.362
	Nervousness	1.000, 23.000	1.000	0.042	0.328
	Sleep Problems	1.308, 30.088	0.561	0.024	0.505

## Data Availability

The data presented in this study are available on request from the corresponding author. The data are not publically available due to ethical restrictions.

## Supplementary Materials

The following are available online at https://www.mdpi.com/article/10.3390/brainsci13010137/s1, Table S1: Post-hoc results of M-SRTT. Results of post-hoc pairwise comparisons of the absolute reaction time of M-SRTT under all stimulation conditions are shown. Table S2. Post-hoc results of M-SRTT. Results of post-hoc pairwise comparisons of the absolute reaction time of M-SRTT between block 6 (random block) to block 5 and 7 (sequence) under stimulation conditions are shown. Table S3. Post-hoc results of M-SRTT. Results of post-hoc pairwise comparisons of the normalised reaction time of M-SRTT under all stimulation conditions are shown. Table S4. Post-hoc results of M-SRTT. Results of post-hoc pairwise comparisons of the normalised reaction time of M-SRTT between block 6 (random block) to block 5 and 7 (sequence) under stimulation conditions are shown. Table S5. Post-hoc results of M-SRTT. Results of post-hoc pairwise comparisons of the errors of M-SRTT under all stimulation conditions are shown. Table S6. Post-hoc results of M-SRTT. Results of post-hoc pairwise comparisons of the errors of M-SRTT between block 6 (random block) to block 5 and 7 (sequence) under stimulation conditions are shown. Table S7. Post-hoc results of M-SRTT. Results of post-hoc pairwise comparisons of the variability of absolute reaction time of M-SRTT under all stimulation conditions are shown. Table S8. Post-hoc results of M-SRTT. Results of post-hoc pairwise comparisons of the variability of absolute reaction time of M-SRTT between block 6 (random block) to block 5 and 7 (sequence) under stimulation conditions are shown. Table S9. Post-hoc results of R-SRTT. Results of post-hoc pairwise comparisons of the absolute reaction time of R-SRTT under all stimulation conditions are shown. Table S10. Post-hoc results of R-SRTT. Results of post-hoc pairwise comparisons of the normalised reaction time of R-SRTT under all stimulation conditions are shown. Table S11. Post-hoc results of R-SRTT. Results of post-hoc pairwise comparisons of the variability of absolute reaction time of the R-SRTT under all stimulation conditions are shown. Table S12. Post-hoc results of R-SRTT. Results of post-hoc pairwise comparisons of the block × condition interaction for the variability of absolute reaction time of the R-SRTT are shown.
