# Supplementary material for: Optimizing the Effect of tDCS on Motor Sequence Learning in the Elderly
Source: Brain Sci. 2023 Jan 12;13(1):137. doi: 10.3390/brainsci13010137 (PMC9857096; doi:10.3390/brainsci13010137)
Supplement: Supplementary file 1 [file brainsci-13-00137-s001.zip › brainsci-2089312-supplementary.pdf]

Optimizing the effect of tDCS on motor sequence learning in the elderly

Ensiyeh Ghasemian-Shirvan[1,2,3], Ruxandra Ungureanu [1,4], Lorena Melo[1,2], Kim van Dun[3], Min-Fang Kuo[1], Michael A. Nitsche[1,5], Raf L.J. Meesen[3,6]

1. Department of Psychology and Neurosciences, Leibniz Research Center for Working Environment and Human Factors, Dortmund, Germany
2. International Graduate School of Neuroscience, Ruhr-University Bochum, Bochum, Germany
3. Neuroplasticity and Movement Control Research Group, Rehabilitation Research Institute (REVAL), Hasselt University, Diepenbeek, Belgium
4. Institute of Cognitive Neuroscience, Ruhr-University Bochum, Bochum, Germany
5. Bielefeld University, University Hospital OWL, Protestant Hospital of Bethel Foundation, University Clinic of Psychiatry and Psychotherapy and University Clinic of Child and Adolescent Psychiatry and Psychotherapy
6. Movement Control and Neuroplasticity Research Group, Department of Movement Sciences, Group Biomedical Sciences, KU Leuven, Belgium

Supplementary materials:

Results of post-hoc comparisons of blocks as well as block and condition interactions are listed in the tables below:

Table S1. Post-hoc results of M-SRTT. Results of post-hoc pairwise comparisons of the absolute reaction time of M-SRTT under all stimulation conditions are shown. Asterisks indicate significant results ($p<0.05$).

Condition	Block	Blocks	*t* value	d.f.	Effect size d	*P* value
**1 mA**	1 (Random)	2 (Sequence)	3.142	23	0.641	0.005*
		3 (Sequence)	1.203	23	0.246	0.241
		4 (Sequence)	1.067	23	0.218	0.297
		5 (Sequence)	2.657	23	0.542	0.014*
		6 (Random)	-1.040	23	-0.212	0.309
		7 (Sequence)	1.164	23	0.238	0.256
		8 (Sequence)	1.078	23	0.220	0.292

**2 mA**	1	2	2.711	23	0.553	0.012*
		3	4.162	23	0.849	<0.001*
		4	2.469	23	0.504	0.021*
		5	2.900	23	0.592	0.008*
		6	-1.320	23	-0.269	0.200
		7	3.100	23	0.633	0.005*
		8	3.334	23	0.680	0.003*
**3 mA**	1	2	3.487	23	0.712	0.002*
		3	2.422	23	0.494	0.024*
		4	2.693	23	0.550	0.013*
		5	1.468	23	0.300	0.156
		6	-1.446	23	-0.295	0.162
		7	1.134	23	0.231	0.269
		8	2.211	23	0.451	0.037*
**Sham**	1	2	3.376	23	0.689	0.003*
		3	2.438	23	0.498	0.023*
		4	2.223	23	0.454	0.036*
		5	3.541	23	0.723	0.002*
		6	-1.079	23	-0.220	0.292
		7	1.447	23	0.295	0.161
		8	2.183	23	0.446	0.039*

Table S2. Post-hoc results of M-SRTT. Results of post-hoc pairwise comparisons of the absolute reaction time of M-SRTT between block 6 (random block) to block 5 and 7 (sequence) under stimulation conditions are shown. Asterisks indicate significant results (p<0.05).

Condition	Block	Blocks	*t* value	d.f.	Effect size d	*P* value
**1 mA**	6 (Random)	5 (Sequence)	4.864	23	0.993	<0.001*
		7 (Sequence)	3.913	23	0.799	0.001*
**2 mA**	6	5	6.179	23	1.261	<0.001*
		7	6.784	23	1.385	<0.001*
**3 mA**	6	5	4.090	23	0.835	<0.001*
		7	3.211	23	0.655	0.004*
**Sham**	6	5	5.909	23	1.206	<0.001*

		7	3.148	23	0.643	0.005*

Table S3. Post-hoc results of M-SRTT. Results of post-hoc pairwise comparisons of the normalised reaction time of M-SRTT under all stimulation conditions are shown. Asterisks indicate significant results ($p<0.05$).

Condition	Block	Blocks	*t* value	d.f.	Effect size d	*P* value
**1 mA**	1 (Random)	2 (Sequence)	3.105	23	0.634	0.005*
		3 (Sequence)	1.555	23	0.318	0.133
		4 (Sequence)	1.412	23	0.288	0.171
		5 (Sequence)	2.709	23	0.553	0.013*
		6 (Random)	-1.225	23	-0.250	0.233
		7 (Sequence)	1.169	23	0.239	0.255
		8 (Sequence)	1.263	23	0.258	0.219
**2 mA**	1	2	2.917	23	0.596	0.008*
		3	4.283	23	0.874	<0.001*
		4	2.627	23	0.536	0.015*
		5	3.030	23	0.619	0.006*
		6	-1.335	23	-0.273	0.195
		7	3.291	23	0.672	0.003*
		8	3.455	23	0.705	0.002*
**3 mA**	1	2	3.752	23	0.766	0.001*
		3	2.627	23	0.536	0.015*
		4	2.850	23	0.582	0.009*
		5	1.622	23	0.331	0.119
		6	-1.625	23	-0.332	0.118
		7	1.179	23	0.241	0.250
		8	2.245	23	0.458	0.035*
**Sham**	1	2	3.354	23	0.685	0.003*
		3	2.576	23	0.526	0.017*
		4	2.489	23	0.508	0.021*
		5	3.709	23	0.757	0.001*
		6	-1.156	23	-0.236	0.259
		7	1.687	23	0.344	0.105
		8	2.423	23	0.495	0.024*

Table S4. Post-hoc results of M-SRTT. Results of post-hoc pairwise comparisons of the normalised reaction time of M-SRTT between block 6 (random block) to block 5 and 7 (sequence) under stimulation conditions are shown. Asterisks indicate significant results ($p<0.05$).

Condition	Block	Blocks	*t* value	d.f.	Effect size d	*P* value
**1 mA**	6 (Random)	5 (Sequence)	5.117	23	1.044	<0.001*
		7 (Sequence)	4.300	23	0.878	<0.001*
**2 mA**	6	5	6.204	23	1.266	<0.001*
		7	6.156	23	1.257	<0.001*
**3 mA**	6	5	4.296	23	0.877	<0.001*
		7	3.696	23	0.754	0.001*
**Sham**	6	5	6.474	23	1.321	<0.001*
		7	3.365	23	0.687	0.003*

Table S5. Post-hoc results of M-SRTT. Results of post-hoc pairwise comparisons of the errors of M-SRTT under all stimulation conditions are shown. Asterisks indicate significant results ($p<0.05$).

Condition	Block	Blocks	*t* value	d.f.	Effect size d	*P* value
**1 mA**	1 (Random)	2 (Sequence)	-0.748	23	-0.153	0.462
		3 (Sequence)	-0.684	23	-0.140	0.501
		4 (Sequence)	-0.387	23	-0.079	0.702
		5 (Sequence)	0.164	23	0.033	0.872
		6 (Random)	-0.328	23	-0.067	0.746
		7 (Sequence)	-0.144	23	-0.029	0.887
		8 (Sequence)	-0.352	23	-0.072	0.728
**2 mA**	1	2	0.666	23	0.136	0.512
		3	1.934	23	0.395	0.066
		4	0.914	23	0.186	0.370
		5	1.206	23	0.246	0.240
		6	-0.115	23	-0.023	0.909
		7	0.800	23	0.163	0.432

		8	0.807	23	0.165	0.428
**3 mA**	1	2	1.319	23	0.269	0.200
		3	2.320	23	0.474	0.030*
		4	1.404	23	0.287	0.174
		5	2.491	23	0.509	0.020*
		6	2.048	23	0.418	0.052
		7	1.160	23	0.237	0.258
		8	1.026	23	0.209	0.315
**Sham**	1	2	-0.735	23	-0.150	0.470
		3	-0.497	23	-0.101	0.624
		4	-0.343	23	-0.070	0.735
		5	-0.659	23	-0.135	0.516
		6	-1.905	23	-0.389	0.069
		7	0.387	23	0.079	0.702
		8	0.617	23	0.126	0.544

Table S6. Post-hoc results of M-SRTT. Results of post-hoc pairwise comparisons of the errors of M-SRTT between block 6 (random block) to block 5 and 7 (sequence) under stimulation conditions are shown. Asterisks indicate significant results ($p<0.05$).

Condition	Block	Blocks	*t* value	d.f.	Effect size d	*P* value
**1 mA**	6 (Random)	5 (Sequence)	0.569	23	0.116	0.575
		7 (Sequence)	0.177	23	0.036	0.861
**2 mA**	6	5	1.797	23	0.367	0.085
		7	1.357	23	0.277	0.188
**3 mA**	6	5	0.000	23	0.000	1.000
		7	-1.101	23	-0.225	0.282
**Sham**	6	5	2.488	23	0.508	0.021*
		7	2.875	23	0.587	0.009*

Table S7. Post-hoc results of M-SRTT. Results of post-hoc pairwise comparisons of the variability of absolute reaction time of M-SRTT under all stimulation conditions are shown. Asterisks indicate significant results ($p<0.05$).

Condition	Block	Blocks	*t* value	d.f.	Effect size d	*P* value
**1 mA**	1 (Random)	2 (Sequence)	0.818	23	0.167	0.422
		3 (Sequence)	-2.015	23	-0.411	0.056
		4 (Sequence)	-2.280	23	-0.465	0.032*
		5 (Sequence)	-1.652	23	-0.337	0.112
		6 (Random)	-3.548	23	-0.724	0.002*
		7 (Sequence)	-2.189	23	-0.447	0.039*
		8 (Sequence)	-3.223	23	-0.658	0.004*
**2 mA**	1	2	-0.665	23	-0.136	0.513
		3	-0.690	23	-0.141	0.497
		4	-2.771	23	-0.566	0.011*
		5	-1.490	23	-0.304	0.150
		6	-3.388	23	-0.962	0.003*
		7	-2.611	23	-0.533	0.016*
		8	-2.415	23	-0.493	0.024*
**3 mA**	1	2	-0.393	23	-0.080	0.698
		3	-2.464	23	-0.503	0.022*
		4	-3.011	23	-0.615	0.006*
		5	-2.749	23	-0.561	0.011*
		6	-3.077	23	-0.628	0.005*
		7	-3.835	23	-0.783	<0.001*
		8	-4.552	23	-0.929	<0.001*
**Sham**	1	2	0.053	23	0.11	0.958
		3	-2.258	23	-0.461	0.034*
		4	-2.172	23	-0.443	0.040*
		5	-2.438	23	-0.498	0.023*
		6	-2.581	23	-0.527	0.017*
		7	-3.202	23	-0.654	0.004*
		8	-3.580	23	-0.731	0.002*

Table S8. Post-hoc results of M-SRTT. Results of post-hoc pairwise comparisons of the variability of absolute reaction time of M-SRTT between block 6 (random block) to block 5 and 7 (sequence) under stimulation conditions are shown. Asterisks indicate significant results ($p<0.05$).

Condition	Block	Blocks	*t* value	d.f.	Effect size d	*P* value
**1 mA**	6 (Random)	5 (Sequence)	1.503	23	0.307	0.146
		7 (Sequence)	0.820	23	0.167	0.420
**2 mA**	6	5	2.211	23	0.451	0.037*
		7	0.925	23	0.189	0.365
**3 mA**	6	5	0.409	23	0.084	0.686
		7	-0.380	23	-0.077	0.708
**Sham**	6	5	1.442	23	0.294	0.163
		7	-0.259	23	-0.053	0.798

Table S9. Post-hoc results of R-SRTT. Results of post-hoc pairwise comparisons of the absolute reaction time of R-SRTT under all stimulation conditions are shown. Asterisks indicate significant results ($p<0.05$).

Condition	Block	Blocks	*t* value	df	Effect size d	*P* value
**1 mA**	1 (Random)	2 (Sequence)	5.402	23	1.103	<0.001*
		3 (Sequence)	4.727	23	0.965	<0.001*
**2 mA**	1	2	5.728	23	1.169	<0.001*
		3	4.805	23	0.981	<0.001*
**3 mA**	1	2	5.110	23	1.043	<0.001*
		3	3.595	23	0.734	0.002*
**Sham**	1	2	7.231	23	1.476	<0.001*
		3	5.707	23	1.165	<0.001*

Table S10. Post-hoc results of R-SRTT. Results of post-hoc pairwise comparisons of the normalised reaction time of R-SRTT under all stimulation conditions are shown. Asterisks indicate significant results ($p<0.05$).

Condition	Block	Blocks	*t* value	d.f.	Effect size d	*P* value
**1 mA**	1 (Random)	2 (Sequence)	5.540	23	1.131	<0.001*
		3 (Sequence)	4.500	23	0.918	<0.001*
**2 mA**	1	2	6.040	23	1.233	<0.001*
		3	5.092	23	1.039	<0.001*

**3 mA**	1	2	5.591	23	1.141	<0.001*
		3	3.519	23	0.718	<0.001*
**Sham**	1	2	7.049	23	1.439	<0.001*
		3	5.831	23	1.190	<0.001*

Table S11. Post-hoc results of R-SRTT. Results of post-hoc pairwise comparisons of the variability of absolute reaction time of the R-SRTT under all stimulation conditions are shown). Asterisks indicate significant results ($p<0.05$).

Condition	Block	Blocks	*t* value	d.f.	Effect size d	*P* value
**1 mA**	1 (Random)	2 (Sequence)	-1.885	23	-0.385	0.072
		3 (Sequence)	-4.717	23	-0.963	<0.001*
	2 (Sequence)	3 (Sequence)	-3.352	23	-0.684	0.003*
**2 mA**	1	2	0.416	23	0.085	0.681
		3	0.317	23	0.065	0.754
	2	3	-0.249	23	-0.051	0.806
**3 mA**	1	2	1.018	23	0.208	0.319
		3	-1.459	23	-0.298	0.158
	2	3	-2.569	23	-0.524	0.017*
**Sham**	1	2	-0.056	23	-0.011	0.956
		3	-2.172	23	-0.443	0.040*
	2	3	-2.687	23	-0.549	0.013*

Table S12. Post-hoc results of R-SRTT. Results of post-hoc pairwise comparisons of the block × condition interaction for the variability of absolute reaction time of the R-SRTT are shown. Asterisks indicate significant results ($p<0.05$).

Block	intensity	intensity	*t* value	d.f.	Effect size d	*P* value
**1** (Random)	Sham	1mA	0.902	23	0.184	0.376
		2mA	-0.190	23	-0.039	0.851
		3mA	-0.257	23	-0.052	0.800
	1mA	2mA	0.546	23	0.111	0.590

		3mA	1.207	23	0.246	0.240
	2mA	3mA	-0.442	23	-0.090	0.663
**2** (Sequence)	Sham	1mA	-0.203	23	-0.041	0.841
		2mA	0.501	23	0.102	0.621
		3mA	0.456	23	0.093	0.652
	1mA	2mA	-0.728	23	-0.149	0.474
		3mA	-0.944	23	-0.193	0.355
	2mA	3mA	0.003	23	0.001	0.998
**3** (Sequence)	Sham	1mA	-0.461	23	-0.094	0.649
		2mA	-2.288	23	-0.467	0.032*
		3mA	0.125	23	0.025	0.902
	1mA	2mA	-2.450	23	-0.500	0.022*
		3mA	-0.462	23	-0.094	0.648
	2mA	3mA	-2.497	23	-0.510	0.020*
